# Syphilis Clinical Complexity: A Dual-Case Study Illustrating Diagnostic Dilemmas and Management Strategies

**DOI:** 10.7759/cureus.65997

**Published:** 2024-08-02

**Authors:** Haritha Subhagan, Annup Balan B, Merlin Moni, Dipu T Sathyapalan, Kiran G Kulirankal

**Affiliations:** 1 Division of Infectious Diseases, Amrita Institute of Medical Sciences, Kochi, IND; 2 Department of Internal Medicine, Division of Infectious Diseases, Amrita Institute of Medical Sciences, Kochi, IND

**Keywords:** cutaneous syphilis, latent syphilis, condyloma lata, late latent syphilis, sexually transmitted infection

## Abstract

Syphilis, a bacterial sexually transmitted infection, poses diagnostic challenges due to its diverse clinical manifestations. This report presents two distinctive cases illustrating the diagnostic dilemmas and management strategies associated with syphilis. The first case describes a male in his early 30s presenting with secondary syphilis and condyloma lata, illustrating the atypical genital lesions that can arise. The second case involves a male in his late 40s with late latent syphilis exhibiting unusual cutaneous manifestations, underscoring the diagnostic complexities of the disease. These cases underscore the importance of healthcare providers remaining vigilant in identifying unusual presentations of syphilis to ensure timely intervention and prevent transmission and complications.

## Introduction

Syphilis is a preventable and curable bacterial sexually transmitted infection (STI), if left untreated can cause serious health issues. It is caused by the pathogenic spirochaete Treponema pallidum subspecies pallidum. The World Health Organization (WHO) estimates that 7.1 million adults between 15 and 49 years old acquired syphilis in 2020 and most infections are asymptomatic or unrecognized [1,2]. Globally, the number of prevalent cases of syphilis was 30.91 million in 1990 and 49.71 million in 2019, with an increase of 60.83% from 1990 to 2019 [3]. A recent study done by Kamat et al. reported a prevalence of 15.04% in India where 24.5% had primary, 44.5% had secondary, 30.5% had latent, and 0.50% had congenital syphilis [4-6].

The diversity in the clinical manifestations of syphilis has been well-established, calling it “the great imitator”. Atypical presentations pose a significant risk of transmission due to diagnostic challenges and treatment delays. Syphilis's capability to mimic common skin diseases, depart from typical clinical patterns, and assume unique forms contributes to this risk. Therefore, healthcare providers must remain vigilant, as prompt and accurate diagnosis is crucial for effective management and prevention of transmission.

In this report, we delve into two distinctive cases of syphilis: secondary syphilis with condyloma lata and late latent syphilis with atypical cutaneous manifestations.

## Case presentation

Case 1

An early 30s male with no known comorbidities presented to the emergency room with complaints of oral and genital rashes for one week. The lesions were of insidious onset which progressively worsened and was associated with burning-type pain. He also complained of on-and-off low-grade fever and generalized tiredness for one-week duration.

**Figure 1:**
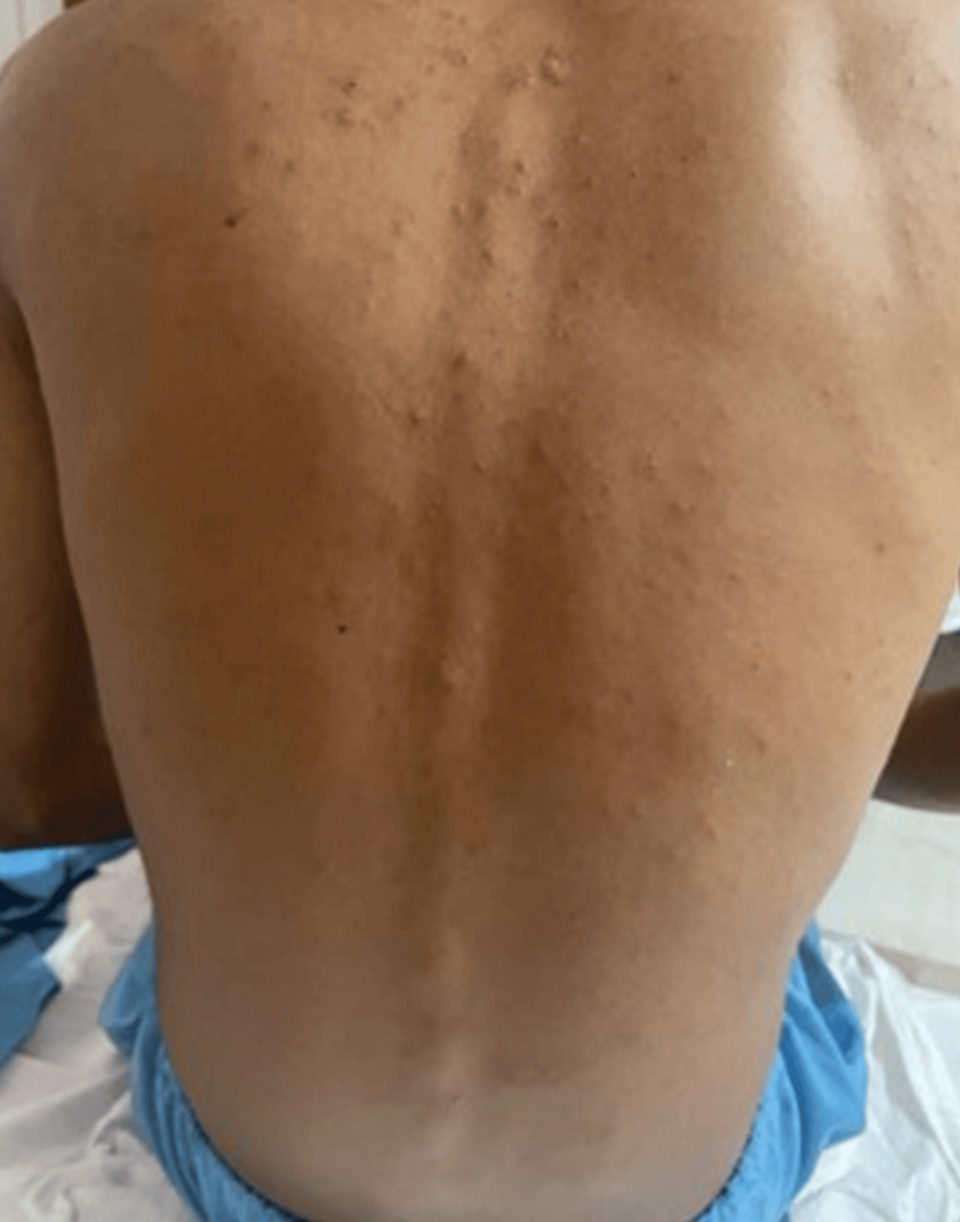
Multiple erythematous papules over the back (Case 1)

**Figure 2:**
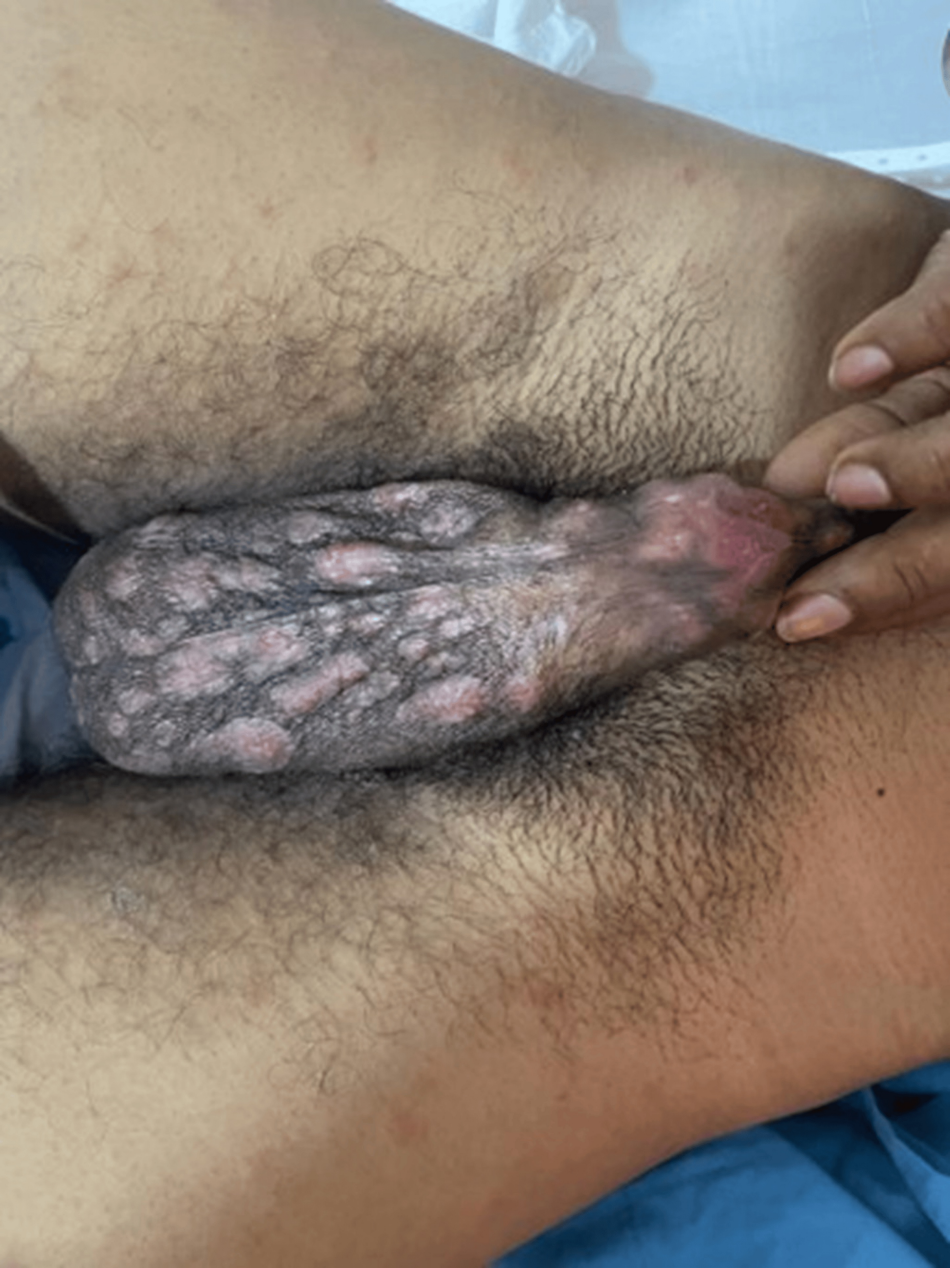
Multiple erythematous well-defined flat-topped moist plaques over the penis and scrotum (Case 1)

Examination of the mouth revealed multiple well-demarcated oral ulcers with surrounding erythema over the lower gingival mucosa, buccal mucosa, base, and lateral aspects of the tongue. There were multiple erythematous papules over his back, trunk abdomen, and arms (Figure 1). There were multiple erythematous well-defined flat-topped moist plaques over the penis and scrotum (Figure 2).

The patient was evaluated and his autoimmune screening (ANA, anti-dsDNA), herpes simplex virus antibodies, vitamin B12, folic acid levels, and HIV status were negative. He was found to have venereal disease research laboratory (VDRL) and Treponema Pallidum hemagglutination assay (TPHA) reactive. VDRL was reported positive with 1:16 titers and TPHA reported positive with 1: 2560 titers.

The patient was diagnosed with secondary syphilis and was administered a single dose of injection Benzathine penicillin 2.4M IU intramuscular as divided doses (1.2M IU on each buttock) and supportive measures. The patient was discharged and reviewed after two weeks where he reported complete recovery of symptoms and rashes.

Case 2

A 48-year-old male working in the Gulf for 15 years who is a known case of systemic hypertension (on Tab.Losartan 50mg OD) and dyslipidemia (on Tab.Atorvastatin 10mg HS) was initially admitted under neurology for evaluation of low back ache of six months duration. MRI spine imaging showed L5-S1 spondylolisthesis.

**Figure 3:**
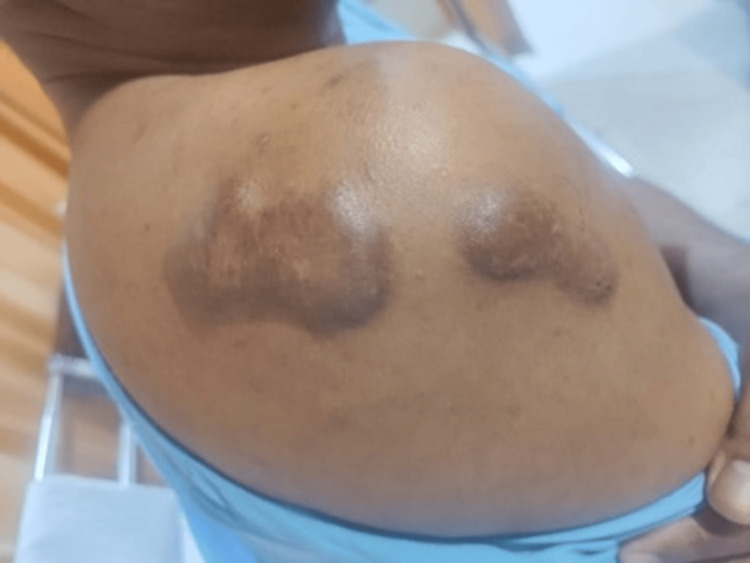
Multiple firm raised hyperpigmented nodules over the right shoulder (Case 1 )

**Figure 4:**
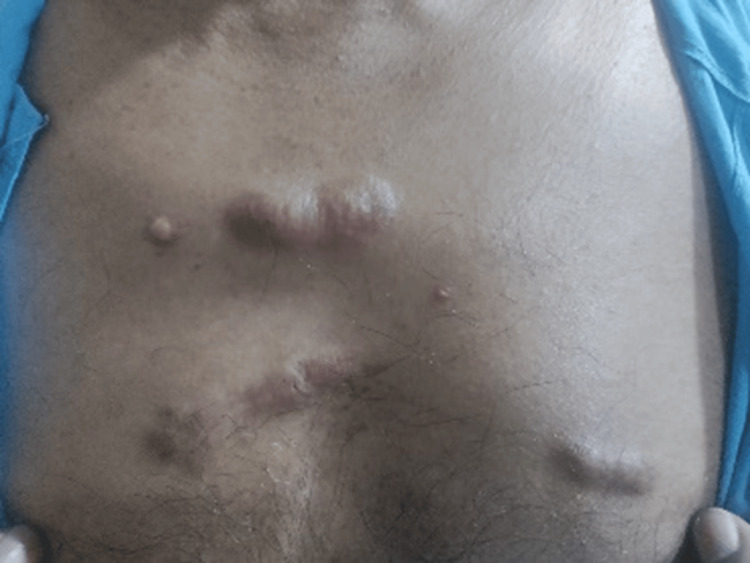
Multiple firm raised hyperpigmented nodules over the anterior chest wall (Case 2)

**Figure 5:**
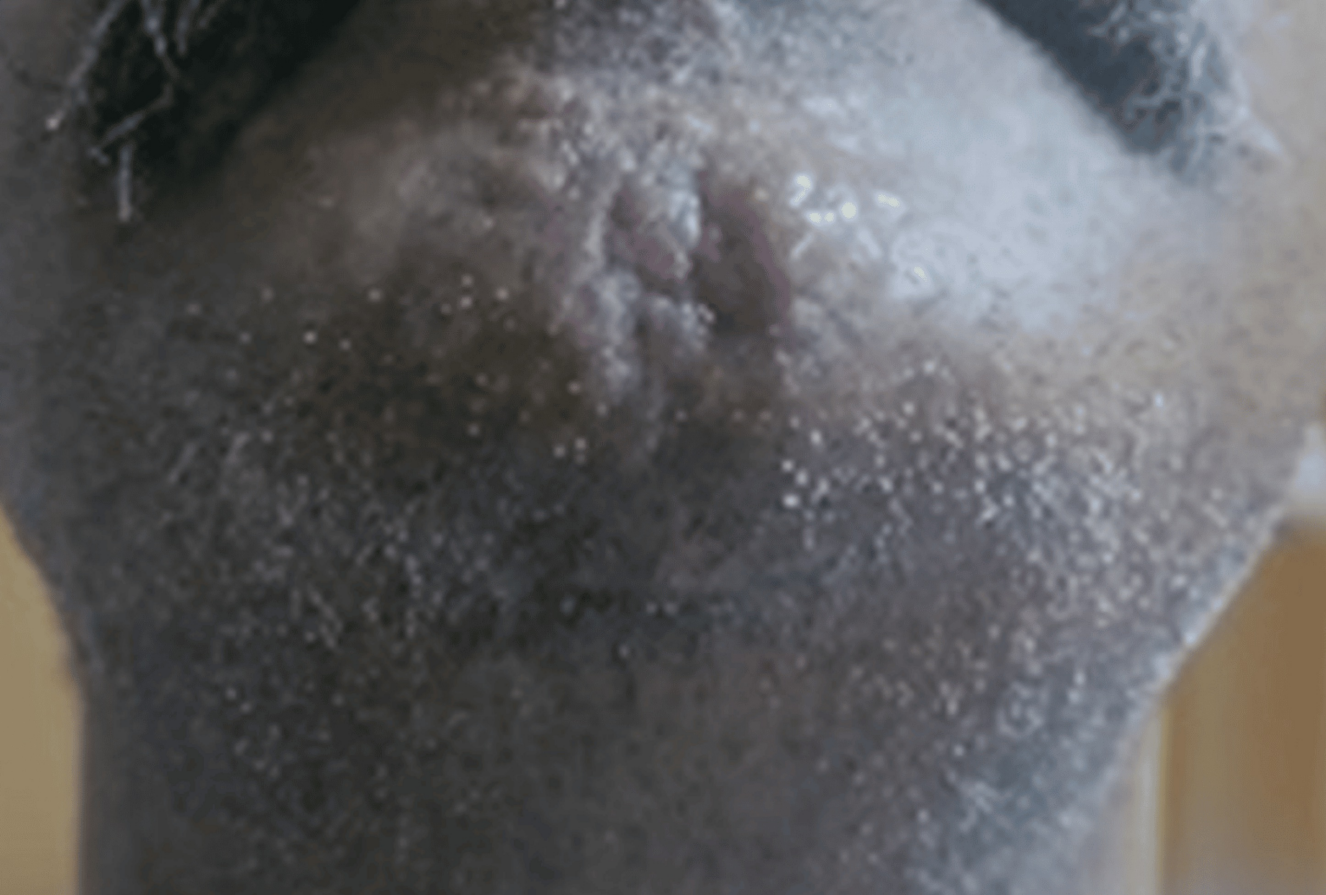
Multiple erythematous papules coalescing to form plaque over the chin (Case 2)

Local examination showed multiple firm raised hyperpigmented nodules over the anterior chest wall and right shoulder (Figures 3, 4) and multiple erythematous papules coalescing to form plaque over the chin and nose (Figure 5).

Systemic examinations were unremarkable. On detailed history taking, he revealed a history of having premarital exposure with multiple women. Skin lesions were present for two years.

He was evaluated and retro status was found to be negative. VDRL and TPHA were sent and positive. VDRL was reported positive with 1:4 titer and TPHA was reported positive with 1:320 titer. Cardiovascular screening with 2D ECHO was done to rule out aortitis, aneurysm formation, aortic valve disease, coronary artery ostial stenosis, myocarditis, and syphilitic gumma and was negative. Ophthalmology consultation was obtained and syphilitic uveitis was ruled out. Lumbar puncture was done and spinal fluid analysis showed protein of 45 mg/dl, glucose of 68 mg/dl (corresponding blood sugar- 81mg/dl), and a cell count of 3/mm^3^ (100% mononuclear), which ruled out the possibility of neurosyphilis. From the spinal fluid, he was diagnosed with late latent syphilis and was administered three doses of Inj. Benzathine Penicillin G 2.4 million units intramuscular given once weekly with which he reported complete recovery after two months of duration.

## Discussion

Treponema pallidum (TP) was identified as the causative organism of syphilis by Fritz Richard Schaudinn and Paul Ehrich Hoffmann in 1905. It is pathogenic only to humans and cannot be cultured in artificial media. It is a slender cork screw-shaped organism whose movements demonstrated by dark field microscopy are pathognomonic.

Almost all cases of syphilis spread through sexual intercourse. While the treponema can pass through intact mucosa, it can more easily penetrate the body through skin breaks brought by sex-related microtraumas. It is rarely transmitted by accidental puncture or blood transfusion. Mother-to-child transmission through the transplacental route causes congenital syphilis.

Primary syphilis often presents as a single non-painful genital ulcer (Chancre) caused by the TP invasion. They can also present as multiple chancres in non-genital areas like fingers, nipples, and oral mucosa and can also appear anywhere in direct contact with the infected site. They are also sometimes associated with lymphadenopathy. The primary lesions usually resolve even in the absence of treatment. If left untreated, primary syphilis may progress to secondary syphilis characterized by systemic involvement.

Clinical manifestations in secondary syphilis arise from hematogenous dissemination of the infection. It may take weeks to months after the primary infection and common manifestations are rashes, lymphadenopathy, and mucocutaneous lesions like mucous patches and condyloma lata. Malaise, alopecia, arthralgia, and pharyngitis are other manifestations. Rarer manifestations include acute meningitis, hepatitis, ulcerative colitis, arthritis, optic neuritis, uveitis, and hypertrophic gastritis. This variability in clinical presentation has led to syphilis being called “the great imitator”.

Our first case presents a patient with secondary syphilis manifesting as condyloma lata. Condyloma lata has been reported to occur in about 6-23% of patients with secondary syphilis [7,8]. Condyloma lata is characterized by flat, moist, wart-like lesions usually seen in the anogenital region. It is highly infectious and is highly suggestive of secondary syphilis.

Without medical intervention, both primary and secondary lesions may heal on their own, putting the patient in an early or latent phase during which no symptoms are present, and this is known as latent syphilis.

Serologic testing is the only method available at this point for detecting the infection. Serologic testing for syphilis is divided into non-treponemal and treponemal and is used for screening, diagnosis, and monitoring of treatment efficacy. Commonly used non-treponemal tests include rapid plasma regain (RPR) and VDRL tests. The sensitivity and specificity of VDRL were 71.6% and 89.5% and those of RPR were 73.5% and 90.5% [9].

Commonly used treponemal tests include TP particle agglutination, fluorescent treponemal antibody absorption test, TP enzyme immunoassay, or chemiluminescent immunoassay and micro hemagglutination assay for TP. Treponemal tests remain positive throughout life. Some patients in the latent phase may progress to tertiary syphilis characterized by cardiovascular syphilis, neurosyphilis, and late benign syphilis. Late latent syphilis can present with atypical cutaneous lesions as in our second case. Mucocutaneous lesions in late latent syphilis can resemble other dermatological conditions leading to diagnostic dilemmas.

In our patient, a very high index of suspicion and proper history-taking may have helped to consider syphilis as a differential. This case emphasizes the need for a high index of suspicion even in the absence of classical clinical symptoms.

Parenteral penicillin remains the recommended first-line treatment for all stages of syphilis [10]. For people with a known allergic tendency to penicillin, alternatives like doxycycline can be used. Treatment for secondary syphilis includes 2.4 million units of benzathine penicillin G administered intramuscularly in a single shot, while latent syphilis requires intramuscular administration of 2.4 million units of benzathine penicillin G once a week for three weeks [11,12]. Several studies have shown the efficacy of parenteral penicillin in the treatment of all syphilis. For instance, Taha et al. (2024) and Thayer et al. (2023) reported comparable clinical improvement [13,14].

## Conclusions

The cases that are being presented show the variety and frequently difficult clinical manifestations of syphilis, highlighting the significance of prompt diagnosis and treatment. While late latent syphilis with atypical cutaneous manifestations emphasizes the diagnostic complications involved with the disease, secondary syphilis presenting with condyloma lata emphasizes the need for caution in diagnosing atypical genital lesions. Post-treatment pictures could not be obtained which is a limitation of the study.

 In the future, initiatives to improve testing, treatment, and awareness will be crucial to reducing the global syphilis burden and enhancing the outcomes of sexual and reproductive health. To do this, cooperation between communities, public health organizations, and healthcare practitioners is essential.
